# Oral health disorders among visually impaired children in South Asian countries: a systematic review

**DOI:** 10.3389/froh.2025.1501120

**Published:** 2025-02-13

**Authors:** Vini Mehta, Siddharthan Selvaraj, Snehasish Tripathy, Neeta Mishra, Sapna Negi, Ankita Mathur, Abedelmalek Kalefh Tabnjh

**Affiliations:** ^1^ Department of Dental Research Cell, Dr. D. Y. Patil Dental College and Hospital, Dr. D.Y. Patil Vidyapeeth, Pune, India; ^2^ Department of Public Health Dentistry, Saveetha Dental College and Hospitals, Saveetha Institute of Medical and Technical Sciences, Saveetha University, Chennai, India; ^3^ Department of the Cariology, Odontology School, Sahlgrenska Academy, University of Gothenburg, Gothenburg, Sweden; ^4^ Department of Applied Dental Science, Faculty of Applied Medical Science, Jordan University of Science and Technology, Irbid, Jordan; ^5^ Dental Research Unit, Center for Global Health Research, Saveetha Medical College and Hospital, Saveetha Institute of Medical and Technical Sciences, Saveetha University, Chennai, India

**Keywords:** oral health, oral diseases, blindness, vision disorders, south Asia

## Abstract

**Background and aim:**

Despite the convergence of visual impairment and poor oral health among children, there is a scarcity of data on the common oral health disorders and their prevalence among children with visual impairments in South Asia. The purpose of this systematic review is to synthesize the existing literature on oral health diseases in visually impaired children in South Asia.

**Methods:**

An exhaustive literature search was carried out in PubMed-MEDLINE, Scopus, Embase and Google Scholar from inception till 31st December, 2024. We included studies if they fulfilled the following criteria: visually impaired children (aged <18 years); assessment using dental examination; children with no other impairment from South Asian regions. Due to the high variability across age groups within the target population, we have synthesized and presented the data in a narrative format.

**Results:**

The search across databases yielded a total of 1,681 studies, out of which 9 studies were included. The prevalence of dental caries was reported ranging from 40% to 98.5%, and dental trauma ranged from 4.62% to 44.28%. High prevalence of malocclusion and gingivitis has also been reported. Quality assessment showed that three studies had medium risk of bias and remaining had a low risk of bias.

**Conclusion:**

In the South Asian region, it is crucial to make special needs dentistry widely accessible, provide caregivers with sufficient dental health information, and ensure dentists receive specialized training to enhance the effectiveness, comfort, and satisfaction of treatment.

**Systematic Review Registration:**

https://www.crd.york.ac.uk/prospero/display_record.php?RecordID=582083, PROSPERO (CRD42024582083).

## Introduction

Visual impairment is an important public health concern, especially among children. Nearly 90 million children under the age of 18 years live with varied degrees of visual impairment worldwide (1). Visual impairment limits the daily lives of visually impaired children, ranging from poor motor skills to challenges in education and healthcare access (2). These restrictions impede their growth and impair their learning abilities to perform routine health and hygiene tasks such as dental hygiene (3).

Managing oral health is a difficult task for visually impaired children since they are unable to correctly perform plaque removal techniques, evaluate their oral health condition, and notice symptoms until the condition worsens (4). As a result, visually impaired children rely heavily on their caregivers or parents for toothbrushing and oral hygiene (3). Despite this, caregivers often face challenges in maintaining the oral health of their visually impaired children due to limited knowledge of proper oral hygiene techniques, a focus on managing overall health, and delays or lack of dental appointments caused by accessibility issues or financial constraints (5).

Numerous studies have shown that children with visual impairments are less likely to brush their teeth regularly and visit the dentist than their sighted peers (6–8). Furthermore, a shortage of specialized dentists and appropriate facilities discourages people from seeking dental care (6–8). These factors increase the likelihood of oral disorders in visually impaired children, including dental caries, periodontal disease, and traumas (9). A global systematic study revealed that children and adolescents with vision impairment experience approximately four times worse oral health outcomes compared to their peers (10). Furthermore, when oral disorders are not addressed in a timely manner, they can lead to speech difficulties and contribute to physical, psychological, and emotional health issues, ultimately affecting the overall quality of life in children (3, 11).

Childhood vision impairment is common in South Asia and is predominantly caused by preventable factors such as congenital cataracts, refractive errors, and retinopathic diseases (12). At the same time, the region contributes to the substantial number of oral health diseases among children, mostly due to inadequate access to oral healthcare services, unhealthy eating habits, and insufficient governmental initiatives directed at oral health (13, 14). Despite the convergence of these two major public health concerns- visual impairment and poor oral health, data are scarce on the common oral health disorders and their prevalence among children with visual impairments in South Asia.

Since oral health programs are often designed with the general population in mind, they frequently overlook the special needs of children with disabilities, such as vision impairments. This highlights the critical need for investigation into the oral health issues experienced by visually impaired children in the region. In the context of this gap, the purpose of this systematic review is to synthesize the existing literature on oral health diseases in visually impaired children in South Asia with the objective to determine the prevalence and types of oral problems. By combining the current information, this review aims to lay the groundwork for future research and policy development, ensuring that visually impaired children receive the attention and care they require to maintain good dental health.

## Methods

### Protocol registration and systematic review guidelines

The protocol for this review was registered and assigned the identification number CRD42024582083 in the PROSPERO database for reviews and dissemination. The preparation of this manuscript adheres to the Preferred Reporting Items for Systematic Reviews and Meta-Analysis Extension (PRISMA) statement, designed for systematic reviews (15, 16).

### Search strategy

An exhaustive literature search was carried out in PubMed-MEDLINE, Scopus, and Embase. The search was conducted from inception till 31st August, 2024 without any language restrictions to identify potential studies. Initially developed for the MEDLINE database, the search strategy was constructed through a mix of medical subject headings (MeSH), free text terms and accessible text terms which was later connected by Boolean terms (Table 1).

**Table 1:** Search terms used for search strategy development.

Keywords	Mesh terms	Key terms
Oral Health	“Oral Health” [Mesh] OR “Mouth Diseases” [Mesh] OR “Oral Manifestations” [Mesh] OR Periodontal Diseases	Oral disease OR oral disorders OR mouth disease OR oral health OR periodontal disease OR teeth problems OR teeth disorders
Visually Impaired	“Visually Impaired Persons” [Mesh] OR “Blindness” [Mesh]	Impaired Persons, Visually OR Blind Persons OR Person, Visually Disabled OR Persons with Visual Impairments OR Blind OR Sight Impairment OR Legal Blindness OR Vision Disability
South Asia	Asia, “Southern” [Mesh]OR “India” [Mesh] OR “Pakistan” [Mesh] OR “Afghanistan” [Mesh] OR “Bangladesh” [Mesh] OR “Bhutan” [Mesh] OR “Maldives” [Mesh] OR “Nepal” [Mesh] OR “Sri Lanka” [Mesh]	Southern Asia OR Asia, South OR British Indian Ocean Territory

The search strategy was customized for each database (Supplementary Table 1). Results were imported into a bibliographic database to facilitate deduplication, while cross-references and Google Scholar were meticulously reviewed.

### Inclusion and exclusion criteria

We included studies if they fulfilled the following criteria:

(1) Population: visually impaired children (aged <18 years) (17);
(2) Intervention: oral health assessment using dental examination;
(3) Comparator: None/children with no impairment.
(4) Outcome: the prevalence of oral disorders like dental caries, periodontitis, etc.
(5) Study settings: South Asian regions
(6) Study type: original studies including longitudinal studies, cohort studies, case-control studies, cross-sectional studies, and controlled clinical trials.

Studies were discarded if they: (1) were review articles, editorial papers, commentaries, letters, or methodological papers to ensure that findings are based on original data rather than interpretation/secondary sources; (2) assessed oral health status based on subjective responses to avoid reporting bias in the data; (3) included visually impaired children with other disabilities, particularly related to motor skills since presence of other disabilities can act as the confounding factors.

### Data extraction

Following a systematic search of relevant articles in databases, the results were uploaded in Rayyan, online software for systematic reviews. Duplicated were identified using automation and removed manually in in Rayyan. Two researchers (SS and ST) began sorting and identifying possibly relevant publications based on their titles and abstracts independently. Any differences between the two investigators about a study's eligibility were settled through lengthy discussions or guidance from an academic expert (VM). The full text of selected abstracts was then downloaded and analyzed for full text eligibility using the inclusion/exclusion criteria. The same two investigators (SS and ST) gathered information separately, using a standardized form in Microsoft Excel (version 2021). The information gathered was compared and summarised to create a single final document from which the analysis was performed.

The information from the included articles was retrieved under the following variables: first author's name, year of publication, the design of the study, study setting, total sample size, and the prevalence of oral health disorders.

### Study quality assessment

The scientific quality of the studies involved in the review was assessed using the Joanna Briggs Institute (JBI) Critical Appraisal Checklists for Prevalence and Analytical Cross-Sectional Studies (18). Two reviewers (SS and ST) independently assessed the quality of the articles, and the final assessment was established by debate with studies scoring more than 70% score considered at low risk of bias, 50%–70% as medium risk of bias and <50% as high risk of bias.

### Data synthesis

Due to the high variability across age groups within the target population, we have synthesized and presented the data in a narrative format.

## Results

**Figure 1:**
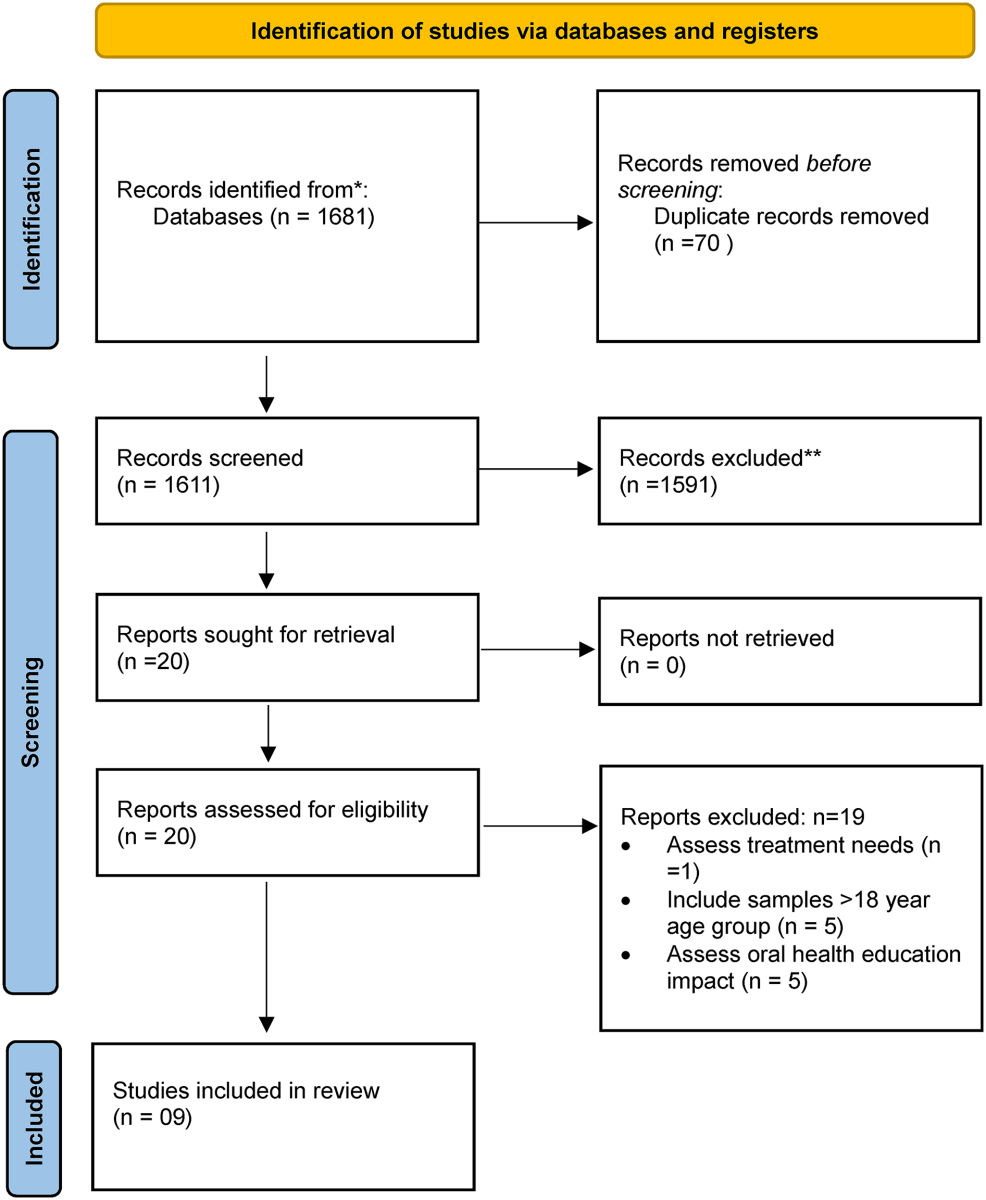
Study selection process. *PubMed-MEDLINE, Scopus, and Embase. **Number of studies excluded manually based on title and abstract Reading.

The search across databases yielded a total of 1,681 studies, out of which 70 were duplicates. After deduplication, remaining 1,611 articles were screened for eligibility based on title and abstract screening. A total of 20 articles were identified as potentially relevant to the topic and were reviewed to determine if they met the inclusion criteria and aligned with the study objectives. A total of 11 articles were further removed due to various reasons as presented in the Figure 1.

Thus, a total of 9 studies were included for data extraction. List of excluded studies is provided in Supplementary Table 2.

The publication year of included studies were between 2010 and 2021. In terms of study setting, All includes studies were from India, conducted in different states *viz.* Tamil Nadu (*n* = 2), Karnataka (*n* = 2), Uttar Pradesh (*n* = 1), Chhattisgarh (*n* = 1), Maharashtra (*n* = 1), Rajasthan (*n* = 1) and Odisha (*n* = 1). Cross sectional study design was used in all included studies. The sample size varied from 85 to 400 with an average sample of 248 ± 126.60. The studies included children aged between 4 and 18 years. The summary of included studies is provided in Table 2.

**Table 2:** Characteristics of the included studies.

Authors/Publication year	Indian state	Study design	Type of population	Sample size	Age range	Data collection tool	Conditions assessed	Authors conclusion
Shetty et al. (3)	Karnataka	Cross-Sectional Study	Visually impaired children	221	6–12	Modified WHO oral health assessment form (1997), Gingival Index, Simplified Oral Hygiene Index	Dental caries, DMFT/deft, gingival status, dental anomalies	suboptimal levels of oral health with majority having high caries prevalence and moderate to severe gingivitis.
Avasthi et al. (19)	New Delhi and Uttar Pradesh	Cross-Sectional Study	Blind children attending special school	350	5–16	DMFT Index, def Index, gingival index, malocclusion, oral health assessment form	Dental caries, gingivitis, malocclusion, dental trauma	Blind children have a high prevalence of dental diseases
Reddy et al. (20)	Tamil Nadu	Cross-Sectional Study	Visually impaired children attending special schools	128	6–15	WHO 1997 criteria based physical examination	Dental caries, DMFT/deft, traumatic experience of the anterior teeth	A higher prevalence of dental caries, and trauma compared to normal children.
Prashanth et al. (21)	Karnataka	Cross-Sectional Study	Visually impaired children	85	8–13	Oral health examination	Dental caries, DMFT/def	Extra care by caretaker regarding oral hygiene can results in reduction of dental caries.
Solanki et al. (22)	Rajasthan	Cross-Sectional Study	Blind school children	354	6–15	oral examination based on WHO basic oral health survey 1997 criteria	Dental caries, DMFT/deft	Higher rates of caries in comparison to the general population
Munot et al. (23)	Chattisgarh	Cross-Sectional Study	Visually impaired children attending special schools	400	6–18	Structured questionnaires, Clinical examination	Dental trauma	Higher risk of multiple TDI
Suresan et al. (24)	Odisha	Cross-Sectional Study	Visually impaired children attending special schools	238	4–15	ADA Type III clinical examination, Oral health assessment form (WHO, 1997), TDI assessment using WHO criteria	dental caries, dental trauma, DMFT/def	High prevalence of dental caries, TDI, and poor oral hygiene found
Bhambere (4)	Maharashtra	Cross-Sectional Study	Visually impaired male students	85	6–13	Oral health examination	DMFT/deft	Caries occurrence was minimal in students with good oral hygiene
Vishnu et al. (25)	Tamil Nadu	Cross-Sectional Study	Visually impaired children	371	5, 12, 15	WHO criteria (Basic Oral Health Survey, 1997)	gingival bleeding, dental caries, dental trauma	High prevalence of gingival diseases and dental Caries in the special need group children

WHO: World Health Organization; DMFT: Decayed, Missed, Filled Teeth; deft: decayed, extracted, filled teeth; TDI: Traumatic dental injury; ADA: American Dental Association.

All studies employed oral health examination to rule out the oral health disorders among the visually impaired children with World Health Organization (WHO) oral health survey 1997 being the most commonly used data collection tool. Dental caries and dental trauma were the most common oral health problem reported.

### Dental caries

The prevalence of dental caries was reported in seven studies (3, 19–22, 24, 25). Of these, four studies reported a prevalence ranging from 40% (20) to 98.5% (3). Two studies specifically examined dental caries in both primary and permanent dentition, with prevalence rates ranging from 15% to 69.4% in primary dentition and 35.2% to 46% in permanent dentition (21, 24). Study conducted in Tamil Nadu compared the prevalence of dental caries across three age groups, reporting 52.6% in the 5-year-old group, 38.5% in the 12-year-old group, and 39.2% in the 15-year-old group (25). The DMFT (Decayed, Missing, and Filled Teeth) and deft (decayed, extracted, and filled teeth in primary dentition) scores were reported in four studies. Reddy et al. (20) reported a DMFT/deft score of 1.1/0.17, Solanki et al. (22) reported 1.1/0.87, and Bhambere et al. (4) reported 0.58/2. Sureshan et al. (24) provided the mean dmft score for primary dentition as 0.48 ± 1.54, and the mean DMFT for permanent dentition as 1.57 ± 2.30.

### Dental trauma

Dental traumatic injuries were the second most commonly reported dental problem across the included studies. Four studies (19, 20, 23, 24) reported the prevalence of dental trauma, which ranged from 4.62% (24) to 44.28% (19). Vishnu et al. (25) observed a higher prevalence of traumatic injuries among visually impaired children in the 12-year-old age group (15.3%) compared to those in the 5-year-old (1%) and 15-year-old (6.2%) groups. According to Munot et al. (23), the most injured teeth were permanent maxillary central incisors. The most common type of injury was enamel-related (53%), followed by 12% affecting enamel and dentine, 12% missing due to trauma, 7% pulp injury, and 5% treated dental injury. Increased overjet and poor lip covering were found to be significantly related to trauma (23).

### Malocclusion

Only three studies (3, 19, 23) reported on tooth malocclusion among visually impaired children. According to Avasthi et al. (19), 2.57% of the children had crowding or spacing issues, 2.28% presented with crossbite, 15.7% had an overjet greater than 2 mm, and 6.85% were categorized under “other” malocclusions. Similarly, Shetty et al. (3) noted that 3.6% children had teeth crowding, 2.2% has teeth spacing and 0.9% had diastema. Munot et al. (23) reported that among the children studied, 240 (60.0%) had an overjet of ≤3.5 mm, while 160 (40.0%) had an overjet greater than 3.5 mm.

### Gingival status

Three studies assessed the gingival status of visually impaired children (3, 19, 25). Avasthi et al. (19) reported a prevalence of gingivitis among 71.53% of the children. Shetty et al. (3) found that 55.6% of the children had moderate gingivitis, 34.4% had severe gingivitis, and 10% exhibited mild gingivitis. Vishnu et al. (25) observed a high prevalence of gingival bleeding, with 86.1% in the 12-year-old age group, compared to 84.5% in the 5-year-olds and 66.2% in the 15-year-old children.

### Quality assessment

Of the studies reviewed, 4 out of 6 had unclear or inappropriate sampling methods, and 5 out of 6 did not provide sample size calculations. However, the majority of the studies (5/6) provided a clear description of the study setting and population. 4/6 studies did not provided details in terms of measuring condition in reliable way (3, 4, 21, 23). Statistical analysis was appropriate in all cases, and response rates were deemed adequate in all studies. Overall, 3 studies had medium risk of bias and remaining had a low risk of bias (Table 3).

**Table 3:** Risk of bias assessment of the included prevalence studies.

Authors	Year	Appropriate sampling frame	Appropriate sampling method	Adequate sample size	detailed description of study setting and population	Data analysis with sufficient coverage of the identified sample	valid methods used for the identification of the condition	condition measured in a standard, reliable way for all participants	appropriate statistical analysis	Adequate response rate
Munot et al. (23)	2017	Yes	No	Unclear	Yes	Yes	Yes	Unclear	Yes	Yes
Vishnu et al. (25)	2021	Yes	Yes	Yes	No	Yes	Yes	Yes	Yes	Yes
Shetty et al. (3)	2010	Yes	Yes	Unclear	Yes	Yes	Yes	Unclear	Yes	Yes
Prashanth et al. (21)	2011	Yes	No	Unclear	Yes	Yes	Yes	Unclear	Yes	Yes
Suresan et al. (24)	2017	Yes	No	Unclear	Yes	Yes	Yes	Yes	Yes	Yes
Bhambere (4)	2017	Yes	No	Unclear	Yes	Yes	Yes	Unclear	Yes	Yes

Both Reddy et al. (20) and Solanki et al. (22) lacked clarity in defining inclusion criteria, did not provide detailed description of study settings and samples and reliable outcome measurement. Both Reddy et al. (20) and Solanki et al. (22) identified confounding factors, while Avasthi et al. (19) did not provide clarity. Only Reddy et al. mentioned strategies for handling confounding factors. Avasthi et al. (19) and Solanki et al. (22) used appropriate statistical analysis, whereas Reddy et al. did not. Overall, Reddy et al. and Solanki et al. had high risk of bias whereas Avasthi et al. has low risk of bias (Table 4).

**Table 4:** Risk of bias assessment of analytical cross-sectional studies.

		Clearly defined criteria for inclusion	Detailed description of study subjects and the setting	exposure measured in a valid and reliable way	Objective, standard criteria used for measurement of the condition	confounding factors identified	strategies to deal with confounding factors stated	outcomes measured in a valid and reliable way	appropriate statistical analysis
Reddy et al. (20)	2011	No	No	Yes	Yes	Yes	Yes	No	No
Solanki et al. (22).	2013	No	No	Yes	Yes	Unclear	No	No	Yes
Avasthi et al. (19)	2010	Yes	Yes	Yes	Yes	Unclear	No	Yes	Yes

## Discussion

Traditional ways of teaching dental hygiene skills, such as demonstrating models or employing plaque-disclosing dyes, are ineffective for children with visual impairments. These children learn how to utilize oral hygiene items mostly through vocal instructions, smells, and touch. However, there is not enough data to determine which methods are most helpful for teaching oral health to visually impaired children. As a result, maintaining oral hygiene for visually impaired children presents significant challenges for both parents/caregivers and dental professionals (26). The aim of this systematic review was to compile current data on the oral health of children and adolescents in South Asia, providing valuable insights for stakeholders involved in planning future preventive and intervention programs.

The studies included reported a high prevalence of dental caries (40%–98%) and elevated DMFT/deft scores. However, variations in the study population, sample size, and clinical assessment procedures contributed to the wide range in caries prevalence. A systematic review found that the prevalence of dental caries varied between 40% and 84% in people with VI (severe blindness) and 11.5%–83% in those without VI (27). Another meta-analysis conducted in the South East Asian region (SEAR) among sighted children found that the overall prevalence of dental caries for permanent dentition was 55% at 5 years old, 45% at 12 years, and 51% at 15 years old, which is lower than the prevalence observed amongst children with visual impairments (28). Similarly, three recent systematic reviews found that visually impaired children had a higher decayed, missing, and filled surface (DMFS/dmfs) index compared to their sighted peers, with the indices increasing in severity based on the degree of blindness (10, 29, 30). A 5-year retrospective study among autistic children reported no significant difference in early childhood caries prevalence between autistic and non-autistic children; nevertheless, treatment modalities differed for both groups (31). This reflects the difficulties children with disabilities have in maintaining oral hygiene and obtaining proper dental treatment. While etiological factors may vary by disability type, our findings underline the need for customized overall health interventions for children with disabilities, including those with visual impairment.Our findings also reveal a high prevalence of gingivitis among this cohort. This is consistent with previous studies (10, 27, 29) which found that visually impaired people had higher gingival indices. Children with visual impairments often have reduced manual dexterity and plaque removal ability due to their limited motor skills and inability to visually detect dental plaque, leading to poor oral hygiene. Furthermore, factors such as infrequent dental checkups, barriers to accessing oral health care, and lack of attention from parents and dental practitioners—whose primary focus is managing the child's disability—also contribute to the poor oral health of visually impaired children (32).

The prevalence of dental trauma was reported to be 4.62%–44.28% in the included studies. Similarly, a systematic review of 27 global studies found that tooth fractures caused by dental trauma affected 33.05% of the people below 20 years of age (33). Silva-Freire et al. (10) in their meta-analysis found that children and adolescents with VI vision impairment were 3.86 times more prone to experience oral trauma than their counterparts. Another systematic study and meta-analysis found that children with visual impairments had 3.84 times greater rates of severe dental injuries than their sighted counterparts (29). The cause of dental trauma in children with visual impairments is a complicated phenomenon termed as visual incompatibility, which impairs their ability to navigate and perceive direction. As a result, they fall in absence of assistance and clash with the objects in their surroundings (34).

Traumatic dental injuries (TDI) affecting dentin/enamel was found to be the most prevalent in our review. When a dental trauma affects enamel or dentin, the situation is generally controllable. Nonetheless, trauma associated with pulpal obliteration/necrosis, fistula and periapical abscess growth, root resorption, and crown discoloration can be concerning because of aesthetic issues. Consistent with our findings, Dash et al. (33) indicated that falls of any kind were the most frequent cause of TDI, followed by malocclusions like overjet and lip incompetency. A systematic analysis revealed that children and adolescents with poor lip coverage have 1.86–2.36 times higher odds of developing TDI, while those with increased overjet have 1.94–3.11 times higher odds (35). The anterior teeth, particularly the incisors, are critical for appearance, speaking, and basic functioning. Problems such as overjet or malocclusion (teeth misalignment) can impede these activities and result in poor dental health and quality of life, particularly in children with visual impairments.

Our findings have several implications for additional research and clinical practice When it comes to dental care, promoting and preventing oral health problems should be a key element in helping visually impaired children's oral health. Significant factors influencing the children's oral health status is the caregiver's gender, educational level, family economic situation, perception of the children's oral hygiene. In addition, two-thirds of caregivers reported challenges in taking their children to the dentist, citing factors such as long appointment wait times, difficulties managing behavioral issues in a dental setting, and dissatisfaction with the quality of dental care provided. These factors contribute to the higher rate of untreated oral health issues in children with special needs. Therefore, it is essential to ensure that special needs dentistry is widely accessible, provide caregivers with adequate dental health information, and equip dentists with specialized training to make treatment more effective, comfortable, and satisfactory (36). Combination approaches, such as specially designed instructional demonstrations, can be used by school-based oral health education programs to improve oral health status, knowledge, and habits (37, 38). The audio tactile performance (ATP) approach has been shown to be an effective tool for providing oral health education and improving the oral hygiene habits of visually impaired children (31, 32, 39). Similarly, innovative initiatives must be created and evaluated.

Our review reveals a significant gap in the literature regarding the oral health of visually impaired children in the South Asian region. Despite our efforts to include studies from across South Asia, we identified relevant research exclusively from India. This underscores the need to prioritize regional oral health research to improve the understanding of key oral health challenges faced by visually impaired children in the SEAR region.

This review has several limitations. Despite employing a comprehensive search strategy across various databases, there remains a possibility that some relevant articles were missed. Additionally, methodological quality issues were identified in the studies, such as the lack of sample representativeness due to convenience sampling, insufficient information about subjects and settings, absence of explanation regarding reliable data collection methods, failure to identify confounding factors, and inappropriate statistical analyses. Future research should aim to address these gaps.

## Conclusion

Despite the limited number of published studies on the oral health of visually impaired children in South Asian regions, this review reveals a high prevalence of dental caries, gingivitis, traumatic dental injuries (TDI), and malocclusion among this vulnerable population. This underscores an urgent need for expanding regional oral health research to better understand and address the unique challenges faced by visually impaired children in the South Asian region.

We strongly advocate for the inclusion of special needs dentistry as a priority within national and regional health agendas. Policymakers must ensure the accessibility of specialized oral healthcare services tailored to this population. Additionally, empowering caregivers through targeted oral health education and equipping dental professionals with specialized training in managing patients with visual impairments are essential steps to enhance the effectiveness and comfort of treatment.

A coordinated approach involving public health initiatives, caregiver education, and professional training can significantly reduce oral health disparities and improve the overall well-being of visually impaired children in South Asia. Immediate action is needed to ensure these children are not left behind in the pursuit of equitable oral health care. By taking immediate action, we can bridge the gap in oral health disparities and improve the quality of life for visually impaired children in the South Asian region.

## Data Availability

The original contributions presented in the study are included in the article/Supplementary Material, further inquiries can be directed to the corresponding authors.
