# Supplementary material for: Oral health disorders among visually impaired children in South Asian countries: a systematic review
Source: Front Oral Health. 2025 Feb 13;6:1501120. doi: 10.3389/froh.2025.1501120 (PMC11865079; doi:10.3389/froh.2025.1501120)
Supplement: Supplementary file 1 [file Table1.docx]

**Supplementary table 1: Search strategy of included databases**

| **Database** | **Search string** | **Results** |
| --- | --- | --- |
| PubMed | ((Oral disease OR oral disorders OR mouth disease OR oral health OR periodontal disease OR teeth problems OR teeth disorders OR dental disorders) AND ("Visually Impaired Persons"[Mesh] OR "Blindness"[Mesh] OR Impaired Persons, Visually OR Blind Persons OR Person, Visually Disabled OR Persons with Visual Impairments OR Blind OR Sight Impairment OR Legal Blindness OR Vision Disability)) AND (Asia, Southern"[Mesh]OR "India"[Mesh] OR "Pakistan"[Mesh] OR "Afghanistan"[Mesh] OR "Bangladesh"[Mesh] OR "Bhutan"[Mesh] OR "Maldives"[Mesh] OR "Nepal"[Mesh] OR "Sri Lanka"[Mesh] OR Southern Asia OR Asia, South OR British Indian Ocean Territory) | 338 |
| Scopus | ( TITLE-ABS-KEY ( oral AND health ) AND TITLE-ABS-KEY ( visually AND impaired ) OR TITLE-ABS-KEY ( blindness ) AND TITLE-ABS-KEY ( children ) OR TITLE-ABS-KEY ( adolescents ) ) | 192 |
| Embase | ('oral health status'/exp OR 'oral health status' OR 'mouth disease'/exp OR 'mouth disease' OR 'tooth disease'/exp OR 'tooth disease') AND ('visually impaired person'/exp OR 'visually impaired person' OR 'blindness'/exp OR 'blindness' OR 'visual impairment'/exp OR 'visual impairment') AND ('child'/exp OR 'child' OR 'adolescent'/exp OR 'adolescent') | 1151 |
|  |  | 1681 |

**Table 2: List of excluded studies**

| **Sl. No.** | **Author** | **Title of study** | **Year** | **Reason for exclusion** |
| --- | --- | --- | --- | --- |
| 1 | Aggarwal et. al. | Assessment of Oral Health Educational Program on oral health status of visually impaired children in New Delhi | 2019 | Assess oral health education impact |
| 2 | Gautam et. al. | Impact of oral health education by audio aids, braille and tactile models on the  oral health status of visually impaired  children of Bhopal City | 2018 | Assess oral health education impact |
| 3 | Dinesh et. al. | Malocclusion and orthodontic treatment need of handicapped individuals in South Canara, India | 2003 | Assess treatment needs |
| 4 | Das et. al. | Effectiveness of a novel oral health education technique in maintenance of gingival health and plaque removal efficacy among institutionalized visually impaired children of Bhubaneswar city: A randomized controlled trial | 2018 | Assess oral health education impact |
| 5 | Syed et. al. | COMPARISON OF CARIES AND GINGIVAL HEALTH STATUS OF DEAF WITH BLIND CHILDREN, KARACHI | 2019 | include >18 |
| 6 | Giri et. al. | Oral Hygiene and Periodontal Status of Visually Impaired Individuals of a Residential School in Eastern Nepal | 2019 | include >18 |
| 7 | Nayak et. al. | Self-Reported Oral Hygiene Habits amongst Visually Impaired Students | 2019 | include >18 |
| 8 | Sanjay et. al. | Dental health status among sensory impaired and blind institutionalized children aged 6 to 20 years | 2014 | include >18 |
| 9 | Shrivastava et. al. | A comparative evaluation of oral health status among institutionalized totally blind children using different methods – A randomized clinical trial | 2022 | Assess oral health education impact |
| 10 | Kumar et. al. | Effect of oral health education and fluoridated dentifrices on the oral health status of visually impaired children | 2012 | Assess oral health education impact |
| 11 | Chopra et. al. | Periodontal Health Status of Visually Impaired Students Attending a Blind School | 2017 | include >18 |
